# Intravenous Infusion of the Normal Salt Solution

**Published:** 1898-08

**Authors:** Valent1ne Taliaferro

**Affiliations:** Atlanta, Ga.; English-American, Peachtree St.


### INTRAVENOUS INFUSION OF THE NORMAL SALT SOLUTION.

BY VALENTINE TALIAFERRO, D. M., ATLANTA, GA.

My chief object in contributing this article is for the purpose of calling to your attention and soliciting your interest in a means of saving life which in certain instances proves the most powerful, the quickest, and often the only means capable of accomplishing any good.

The intravenous injection of the normal salt solution has largely taken the place of direct blood transfusion in the treatment of many conditions in which blood was formerly used, and has proven equally as efficacious. Being decidedly less dangerous in its use, and more easily procured in such quantities as are desired, it is practically free from the objections always urged against transfusion.

The dangers and difficulties of this operation have been greatly overestimated, and it is perhaps on this account that many are deterred from its use where it is plainly indicated. Even though it were dangerous and difficult, we should not fear to use it in those extreme cases where heroic measures are always justifiable.

So many and so brilliant have been the results obtained by this method, literally bringing back into life those dying from great hemorrhage or profound shock, that it must no longer be looked upon as a curious experiment, but as an established therapeutic measure of power and certainty.

After one has bled to death, over one-half of the blood still remains in the vessels, which is sufficient to sustain life if it can be kept in circulation. Where blood is lost the vaso-motor nerves cause the vessels to contract in proportion to the amount lost, thereby keeping up the normal pressure. Beyond a certain point this compensation fails with great suddenness. By increasing the bulk of the circulating fluid sufficiently to produce the proper tension in the vessels, the heart will resume and continue its work. The hot saline solution will powerfully stimulate the heart and contract the arteries. In pronounced shock without hemorrhage, the blood accumulates in the dilated veins, principally of the abdomen, the arterial pressure being lowered in the same way as in hemorrhage, the salt solution acting as in hemorrhage.

It is chiefly in the treatment of profuse hemorrhage and profound shock that the salines have their greatest field of usefulness, acting here with truly magical effect. Success so surely follows this method in hemorrhages from any cause which are so profuse as to immediately threaten life, that he is guilty of gross neglect who fails to offer this chance of recovery to any who are in extremis.

It is the obstetrician and gynecologist who are so often called upon to combat those sudden, profuse and rapidly fatal hemorrhages, to whom this life saving means should most strongly appeal. How many poor women have bled to death! I dare say there is not one among us who has not seen such an ending, standing in the presence of this grim and deadly ememy, who has not felt powerless to loosen its dreaded grasp. With the means now at our command we can fill and contract the emptied vessels, stimulate the heart, keep up the circulation, stop the bleeding, and restore to safety those who are in imminent peril.

In prolonged surgical operations and accidents, it is usually not the hemorrhage which we dread, for this can generally be controlled; but it is the inevitable shock which is so often rapidly fatal. There is nothing known to us which is so potent in its relief, so rapid in its action, or so certain in its effect as large infusions of hot salt solution.

Some other grave and interesting conditions which are amenable to saline infusion I will briefly mention. A usual fatal complication or termination of diabetes mellitus is diabetic coma. More than one-half the cases of diabetes terminate fatally in this condition. The coma is generally brought about suddenly by excessive exertion, great mental strain or nerve-depression. The two theories most generally accepted upon which this symptom is immediately dependent, are the want of alkalinity of the blood, and the absorption from the alimentary canal of toxic substances. The presence of acetone in the blood is, perhaps, another cause. But setting theory aside, certain it is that in the coma of diabetics there is great decrease in the alkalinity of the blood, and by corrrecting this abnormal condition we are favoring, to the best of our ability, a prolongation of life. By restoring to consciousness and staying the advent of death, which the infusion will always do, even for a short while, sufficient time may possibly be gained to re-establish renal activity, and thereby avoid a fatal termination. Here it is the saline infusion upon which we must depend, acting both as a local and systemic diuretic, and increasing the alkalinity of the blood. In profound diabetic coma we can hope for but little permanent good from this measure, but even here we can bring our patients into a state of consciousness, lasting from a few minutes to hours, and, occasionally, with permanent benefit. It is in coma of milder degree, and especially in threatened coma, that the saline acts most satisfactorily, but it must be used early and in large and repeated quantities.

In the recent epidemics of cholera, in those cases of rapid waste and collapse from exhaustive diarrhœa, saline infusions have been extensively used and with great benefit. In excessive diarrhœa, persistent vomiting and acute anæmia, infusion, if not deferred too long, offers every hope of success.

Recently, in France, great interest has been revived in this procedure by the experiments of M. Claisse, Lejars, Duret and others. By what is known as "lavage of the blood," it is claimed that toxic principles can be rapidly removed from the circulating fluid and grave septic conditions successfully combated. Certain infectious diseases are being treated by this method. We await their further reports with great interest.

The best method of performing infusion is of no little importance. There are many methods and instruments now in use for which superior advantage is claimed. The simplest and safest is the one which deserves our attention, the one to which we should give preference. The Aveling apparatus I find the best for both transfusion and infusion. Dr. Aveling invented his simple instrument in 1864, and with little modification it has remained the best to the present day. It consists of a rubber syringe similar to the Davidson, but made in one piece without valves or joints. Each end is provided with a silver ranula and stop-cock. By knowing the capacity of the bulb the amount of fluid infused can be easily estimated. With this instrument the speed with which the fluid is introduced can be accurately regulated and the tension of the circulation readily determined. All of these are important points, and with no other instrument can they be so precisely regulated.

A prominent vein at the bend of the arm, preferably the median basilic, is usually chosen, though I prefer to go below the elbow, thus allowing the patient to bend the arm at will after the operation without pain or danger to the incision. Through an incision half an inch in length, the vein can be sufficiently exposed. Great care should be taken not to lose any of the patient's blood, and to prevent this a ligature is passed around the distal end of the vein and tied in a *single knot*. But one ligature is necessary, the valves preventing the backward flow of the blood. A longitudinal incision is made with a sharp-pointed bistoury into the vein, in proportion to the size of the canula used; I prefer this to the V-shaped incision, as there is less danger of entirely severing the collapsed vein and the incision is more easily united. The syringe should be completely filled before introducing in the vein, and every precaution taken to prevent the entrance of air. When the canula is removed a continuous suture closes the skin. Before the stitches are tightened, the ligature, which is tied in only one knot, is easily slipped off.

When we remember that with a saline solution the volume of blood can be increased to six times its normal amount without producing death from plethora, it should encourage us to use, without fear, a sufficient quantity of fluid to at least fill the depleted vessels. Four or five pints at a time are required in the worst cases, rarely less than two. A few ounces are of no avail, but the quantity must be determined at the time of the operation by the effect produced. By the resistance to the fluid infused, the tension of the circulation is communicated to the hand which presses the bulb, enabling us in this way to avoid over-distention. The pulse also affords valuable information and should be carefully observed during the operation.

Plain water, even when in small quantities, quickly exerts a very deleterious action upon the whole of the circulating fluid, and will rapidly produce death. The blood plasma is diluted and soon loses its normal alkalinity. By its physical action on the corpuscles they become swollen and pale, the hemoglobin is dissolved out, and their vitality lost. One drachm of sodium chloride to the pint of sterilized water, which has been strained, boiled and cooled to 110° or 115° F., is all that is necessary. The heat is an important factor and should never be omitted.

I have performed this operation eight times, have assisted in several others, and have not yet seen an evil result follow. Nor have I seen a single case where the remedy failed to accomplish some good. Like many other measures which are considered difficult in their administration, or dangerous in their use, it is often delayed until too late to hope for success. If used in time, before the vital functions are damaged beyond repair, we can in the great majority of cases be assured of success.

I could detail, without end, cases of hemorrhage and shock reported by our ablest surgeons, which have been saved from certain death by the prompt infusion of the salt solution. Those who have tried it are unanimous in giving their hearty support. My own experience has been most gratifying, and I rely upon it when all else fails.

English-American, Peachtree St.

